# A novel promising diagnosis model for colorectal advanced adenoma and carcinoma based on the progressive gut microbiota gene biomarkers

**DOI:** 10.1186/s13578-022-00940-1

**Published:** 2022-12-26

**Authors:** Junfeng Xu, Zhijun Zheng, Lang Yang, Ruoran Li, Xianzong Ma, Jie Zhang, Fumei Yin, Lin Liu, Qian Xu, Qiujing Shen, Xiuping Shen, Chunyan Wu, Jing Liu, Nan Qin, Jianqiu Sheng, Peng Jin

**Affiliations:** 1 Senior Department of Gastroenterology, The First Medical Center of Chinese PLA General Hospital, Beijing, 100853 China; 2 Realbio Genomics Institute, Shanghai, 201114 China; 3 Department of Gastroenterology, The Seventh Medical Center of Chinese PLA General Hospital, Beijing, 100700 China; 4 Graduate School, Chinese PLA General Hospital, Beijing, 100853 China; 5 Tenth People’s Hospital of Tongji University, Shanghai, 200072 China

**Keywords:** Colorectal cancer, Advanced adenoma, Metagenomic sequencing, Progressive microbiota gene markers, Diagnosis model

## Abstract

**Background:**

Colorectal cancer (CRC), a commonly diagnosed cancer often develops slowly from benign polyps called adenoma to carcinoma. Altered gut microbiota is implicated in colorectal carcinogenesis. It is warranted to find non-invasive progressive microbiota biomarkers that can reflect the dynamic changes of the disease. This study aimed to identify and evaluate potential progressive fecal microbiota gene markers for diagnosing advanced adenoma (AA) and CRC.

**Results:**

Metagenome-wide association was performed on fecal samples from different cohorts of 871 subjects (247 CRC, 234 AA, and 390 controls). We characterized the gut microbiome, identified microbiota markers, and further constructed a colorectal neoplasms classifier in 99 CRC, 94 AA, and 62 controls, and validated the results in 185 CRC, 140 AA, and 291 controls from 3 independent cohorts. 21 species and 277 gene markers were identified whose abundance was significantly increased or decreased from normal to AA and CRC. The progressive gene markers were distributed in metabolic pathways including amino acid and sulfur metabolism. A diagnosis model consisting of four effect indexes was constructed based on the markers, the sensitivities of the Adenoma Effect Index 1 for AA, Adenoma Effect Index 2 for high-grade dysplasia (HGD) adenoma were 71.3% and 76.5%, the specificities were 90.5% and 90.3%, respectively. CRC Effect Index 1 for all stages of CRC and CRC Effect Index 2 for stage III–IV CRC to predict CRC yielded an area under the curve (AUC) of 0.839 (95% CI 0.804–0.873) and 0.857 (95% CI 0.793–0.921), respectively. Combining with fecal immunochemical test (FIT) significantly improved the sensitivity of CRC Effect Index 1 and CRC Effect Index 2 to 96.7% and 100%.

**Conclusions:**

This study reports the successful diagnosis model establishment and cross-region validation for colorectal advanced adenoma and carcinoma based on the progressive gut microbiota gene markers. The results suggested that the novel diagnosis model can significantly improve the diagnostic performance for advanced adenoma.

**Supplementary Information:**

The online version contains supplementary material available at 10.1186/s13578-022-00940-1.

## Background

Colorectal cancer (CRC) is a major public health concern worldwide and ranks third among all cancers in terms of incidence and second in terms of mortality [1]. It has now become the third most commonly diagnosed cancer and the fourth most common cancer cause of death in 2016 across China [2]. Accumulating evidence suggests that microbial dysbiosis in the human gut may be an important environmental factor in CRC, and several species of bacteria have garnered attention owing to their associations with colorectal carcinogenesis [3–8].

Recently, it is shown that the fecal microbiome could be a source of targeted non-invasive biomarkers for colorectal adenoma and cancer. Yu et al. reported the first successful cross-ethnic validation of metagenomic gut microbial markers for CRC [9]. Further another study showed that *Fusobacterium* species could serve as biomarkers to differentiate patients with CRC and advanced adenoma (AA) from controls [10]. A novel bacterial marker m3 for the non-invasive diagnosis of colorectal adenoma has been identified and evaluated [11].

It is well known that the occurrence of CRC is a multi-gene, multi-step process caused by the interaction of environmental factors with its genome. In most cases, the disease begins as a benign adenomatous polyp, which develops into advanced adenoma with high-grade dysplasia and then progresses to invasive cancer [12]. Therefore, it is clinically valuable to find non-invasive progressive fecal microbiota biomarkers that can reflect the dynamic changes of the disease from normal to adenoma and CRC by continuously increasing or decreasing. Nevertheless, reports of such markers are limited at present.

Here, we performed a metagenomic analysis of samples from colorectal advanced adenoma and carcinoma patients with different stages and control subjects and identified the progressive microbiota biomarkers. Then, we constructed a diagnosis model based on these markers and evaluated its performance as a diagnostic tool for AA and CRC.

## Methods

### Sample collection and public data source

Stool samples were collected from individuals undergoing colonoscopy at the Seventh Medical Center of Chinese PLA General Hospital. The cohort included individuals with digestive symptoms to outpatient gastroenterology clinics, as well as asymptomatic individuals aged 50–85 years. The exclusion criteria were: (1) use of antibiotics within the past 3 months; (2) vegetarian diet; (3) surgery or invasive procedure within the past 3 months; (4) IBD; or (5) history of any cancer. After the written informed consent, each individual was asked to provide a stool sample before bowel preparation. The stool samples were divided and stored at – 80 ºC within 1 h for further analysis.

The public data of samples downloaded from three previous studies,128 samples were from Yu et al. (PRJEB10878), 156 samples were from Feng et al. and 199 samples were from Zeller et al. (ERP005534) [9, 13, 14]. All the sequences of the 483 samples were obtained using an Illumina HiSeq platform with the PE100 sequencing strategy. The public data included 616 samples in validation cohort 1, which were downloaded from the DDBJ Sequence Read Archive (DRA006684 and DRA008156). (see Additional file 2: Table S2) [15]. The 616 samples contained 291 control subjects (NC-V group), 67 multiple polypoid adenomas with low-grade dysplasia (MP group), 73 high-grade dysplasia (HD-V group), and 111 CRC at stage I/II, and 74 CRC at stage III/IV. The public data included 140 samples in validation cohort 3 from Andrew et al. (PRJNA447983) [16]. In this study, advanced adenoma (AA) was defined as adenoma with a ≥ 25% villous component, a diameter ≥ 10 mm, or adenoma with high-grade dysplasia. The stages of invasive adenocarcinoma were determined from the surgically resected specimens with the use of the American Joint Committee on Cancer (AJCC) staging system.

### DNA extraction and whole-genome shotgun sequencing

DNA was extracted from each frozen fecal sample using a QIAamp Power Fecal DNA Kit (Qiagen str. 140724, Hilden, Germany) according to the manufacturer’s protocols. The concentration of the extracted DNA was measured with Qubit, and the molecular size was estimated by agarose gel electrophoresis. To find progressive markers that satisfied the requirement of continuous increase or decrease from controls to advanced adenoma and CRC across different cohorts or different sequencing platforms, a HiSeq XTen platform (Illumina) was used in the samples of discovery cohort 1, while sequencing libraries were generated with a TruSeq DNA Sample Prep v2 Kit (Illumina, Inc., San Diego, CA, USA). However, whole-genome shotgun sequencing of the samples of discovery cohort 2 was carried out on a BGISeq-500 platform, and sequencing libraries were generated with an MGIEasy DNA Sample Pre Kit. The DNA library quality was both confirmed with a Qubitand Agilent 2100.

### Criteria for CRC samples in discovery cohort 1 

**Fig. 1:**
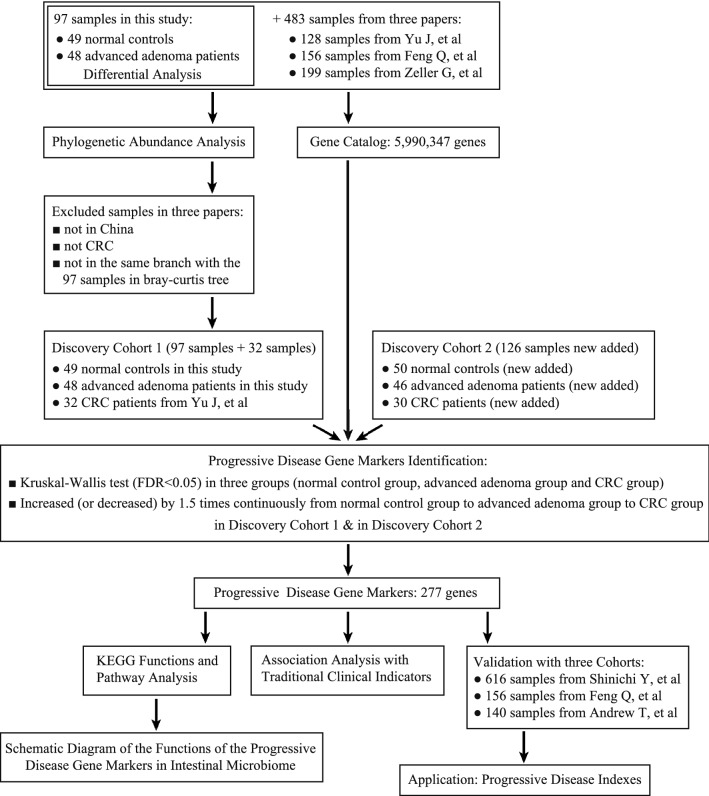
Study design and flow diagram

After an analysis of the difference in intestinal microbiome between the AA group and the controls group (discovery cohort 1), we aimed to further investigate the richness of these differential markers in the CRC group. However, in discovery cohort 1, we did not collect CRC samples, so we selected some of them from the downloaded public raw data. We set the criteria for selecting CRC samples in discovery cohort 1 as follows: (1) sequencing platform: Illumina HiSeq PE100; (2) region of origin of the samples: China; (3) state: CRC; (4) samples in the same branch as the 97 samples from discovery cohort 1 in the Bray–Curtis tree (see Additional file 1: Fig. S3). Finally, we selected 32 CRC samples from the previous three studies (Fig. 1).

### Quality control of metagenomic sequences and phylogenetic abundance profiling

Metagenomic reads with more than 50% low-quality bases (quality ≤ 20) or more than five ‘N’ (bases not identified) were excluded. Furthermore, the reads that could be mapped to the human genome (GRCh38) through SOAP alignment (v2.21) were discarded. The remaining reads in each sample were considered to be high-quality reads.

The sequencing reads of all of the samples in discovery cohort 1 were aligned to reference genomes collected from NCBI and HMP. Phylogenetic abundance profiling was performed as described by Wen et al. [17]*.* A Bray–Curtis tree analysis of the 97 samples collected in this study and 300 disease case samples from previous studies was constructed using the vegan R package based on genus abundance profiling (see Additional file 1: Fig. S3).

### Gene catalog construction gene abundance profiling

All the reads of the samples in discovery cohort 1 were assembled by SOAPdenovo (v2.04). Four k-mers 51, 55, 59, and 63 were used, and the N50-highest k-mer was chosen. After the scaffold was assembled, the scaffolds were split into ‘scaftigs’ by removing ‘N’ bases, and scaftigs of length less than 500 bp were excluded. The genes were predicted from the remaining scaftigs by MetaGeneMark (prokaryotic GeneMark.hmm version 2.8), and the genes with a length less than 100 bp were filtered. All the genes were clustered using CD-HIT (v4.5.7) to construct a non-redundant gene catalog. Gene abundance profiling was performed as described by Wen et al. [17]*.* The protein sequences in the gene set were aligned to the Kyoto Encyclopedia of Genes and Genomes (KEGG) protein database using BLAST with parameters-p-rot-min score = 60-out = blast8.

### Fecal immunochemical test

The FIT was performed using the Eiken OC-Sensor (Eiken Chemical Co., Ltd., Tokyo, Japan) to quantify the fecal hemoglobin. Stool samples with a hemoglobin greater than 20 µg/gm of dry stool (equal to 100 ng/mL of the buffer) were considered positive according to the manufacturer’s instructions.

### The formula of the diagnosis model with different effect indexes

Dichotomy thought and the standard least-squares method were used for the modeling of each effect index with JMP 10 software. For the Adenoma Effect Index1 (AEI1), the initial scores of the controls were 1, and the initial scores of samples with advanced adenoma and above (high-grade dysplastic adenomas and all CRC patients) were 50. For Adenoma Effect Index2 (AEI2), the initial scores of all the controls were 1, and the initial scores of the high-grade dysplastic adenomas and above (all the CRC patients) were 50. For CRC Effect Index1 (CEI1), the initial scores of all the controls were 1, and the initial scores of stage I/II CRC patients and above (stage III/IV CRC patients) were 50. For CRC Effect Index2 (CEI2), the initial scores of all the controls were 1, and the initial scores of stage III/IV CRC patients were 50. Then, the training of the model was carried out by fitting the scores through the standard least-squares method. After fitting, if the majority of the scores were unsatisfactory, the good scores were picked to replace 1 or 50, and the training was carried out again until there was little increase in sensitivity or specificity. Finally, we obtained the following formulas for AEI1, AEI2, CEI1, and CEI2.

$${\text{AEI1}} = \sum\limits_{i = 1}^{277} {e_{i} \cdot \{ \ln [abun(x_{i} )} + {0.0000000001}] + {23}{{.026\} }} + b_{e} ,$$

where *e*_*i*_ represents the parameters of AEI1 (see Additional file 2: Table S8), *x*_*i*_ represents the gene IDs, *b*_*e*_ is the intercept of AEI1, and *abun*(*x*_*i*_) is the relative abundance of the progressive disease gene markers in the intestinal microbiome in each sample (see Additional file 2: Table S9).

$${\text{AEI2}} = \sum\limits_{i = 1}^{277} {f_{i}} \cdot \{ {\rm ln}[abun(x_{i} ) + 0.0000000001] + 23.026\} + b_{f} ,$$

where *f*_*i*_ represents the parameters of AEI2, and *b*_*f*_ is the intercept of AEI2.

$${\text{CEI1}} = \sum\limits_{i = 1}^{277} {g_{i} \cdot \{ {\rm ln}[abun(x_{i} )} + 0.0000000001] + 23.026\} + b_{g} ,$$

where *g*_*i*_ represents the parameters of CEI1, and *b*_*g*_ is the intercept of CEI1.

$${\text{CEI2}} = \sum\limits_{i = 1}^{277} {h_{i}} \cdot \{ {\rm \ln} [abun(x_{i}) + 0.0000000001] + 23.026\} + b_{h} ,$$

where *h*_*i*_ represents the parameters of CEI2, and *b*_*h*_ is the intercept of CEI2.

### Combinations of FIT with CEI1 and CEI2

The formula for the combination of FIT and CEI1 is:

$score1 = 23.710037 + {4}{\text{.082365}} \times {\text{FIT}} + {0}{\text{.147425}} \times {\text{CEI1}}$, with a cutoff of 28.55 (see Additional file 2: Table S10).

The formula of the combination of FIT and the CEI2 is:

$score2 = - 0.87127 + {21}{\text{.769347}} \times {\text{FIT}} + {0}{\text{.332905}} \times {\text{CEI2}}$, with a cutoff of 22.75 (see Additional file 2: Table S10).

### Statistical analysis

The differential species-level markers between controls and patients with advanced adenoma in discovery cohort 1 were tested by Wilcoxon rank-sum test, and a *P*-value of 0.05 or less was considered statistically significant. These differential species markers were further filtered using the mRMR algorithm (side-channel attack R package), and the top 100 markers were used for the next step. Finally, the 25 markers with the highest MCC were selected to build an SVM classifier (the e1071 R package). The progressive disease markers were identified by the Kruskal–Wallis test with the threshold FDR < 0.05 and the median abundance values of the control group, advanced adenoma group, and CRC group should be increased (or decreased) by more than 1.5 times continuously in the discovery cohort 1, and the markers should also be through the same requirements in discovery cohort 2 (see Additional file 2: Table S3). Areas under the receiver operating characteristic curves (AUCs) were constructed using the pROC R package. Spearman correlation coefficients by using an R script were calculated in the abundance profiling of the progressive microbiota biomarkers and the clinical information of subjects (see Additional file 2: Table S1). If the clinical data was ‘NA’, the abundance profiling of the sample was eliminated from the abundance profiling table. A heatmap of the spearman correlation coefficients was constructed using R packages g-plot and RColorBrewer. For a comprehensive description of the details of the establishment of the diagnostic model, see online supplementary methods.

## Results

### Patient cohorts and differences in gut microbiome between advanced adenoma and controls

**Fig. 2:**
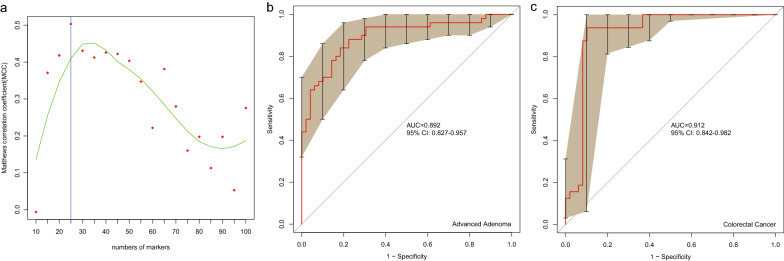
**a** The Matthews correlation coefficient analysis showed the best markers in the SVM modeling for AA. ROC curves of the 25 species markers for the discrimination between advanced adenoma (**b**) and colorectal cancer (**c**) and control subjects in the discovery cohort 1.

The mean age of the 227 subjects (30 CRC, 98 advanced adenomas, and 99 control subjects) from Beijing in the discovery cohort was 60.0 years (see Additional file 2: Table S1). We carried out a metagenomic study in a cohort including 48 patients with AA and 49 control subjects. The genus and species composition of the gut microbiome differed between the 2 groups (see Additional file 1: Fig. S1). At the genus level, *Blautia*, *Bifidobacterium*, *Dorea*, *Sutterella*, and *Tyzzerella* were enriched in patients with AA (*p* < 0.05, see Additional file 1: Figs. S1a, S2a). *Parabacteroides* and *Coprobacter* were enriched in controls (*p* < 0.05, see Additional file 1: Fig. S1b, S2a). At the species level, specific gut bacteria including *Bilophila wadsworthia*, *Eubacterium* sp. CAG 76, *Ruminococcus torques*, *Bifidobacterium longum*, *Dorea longicatena*, *Blautia obeum*, and *Dorea formicigenerans* were enriched in patients with AA (*p* < 0.05, see Additional file 1: Fig. S1c, S2b), whereas other bacteria as *Bacteroides* sp. 4_1_36, *Bacteroides* sp. D20, *Parabacteroides merdae*, *Bacteroides finegoldii*, *Bacteroides* sp. UW, *Alistipes* sp. AL_1 and *Alistipes onderdonkii* were enriched in controls (*p* < 0.05, see Additional file 1: Fig. S1d, S2b). We selected 25 biomarkers at the species level (*p* < 0.05, see Additional file 2: Table S4) that could significantly discriminate advanced adenoma patients from control subjects (AUC = 0.892, 95% CI 0.827–0.957, Fig. 2a, b).

### Phylogenetic abundance analysis and gene catalog construction

We downloaded 483 samples (fecal samples, microbiome, Illumina HiSeq platform) from three previous studies. All downloaded samples and 97 samples (48 AA and 49 controls) collected in this study were used in phylogenetic abundance analysis and to construct a gene catalog. The gene catalog contained 5990347 genes. We further found that CRC samples from Austria, France, Germany, Hong Kong, and Beijing were distributed in different branches by using a Bray–Curtis tree analysis (see Additional file 1: Fig. S3). Furthermore, following the criteria set in the previous method, we selected 32 samples from the three studies as the CRC group in discovery cohort 1 (Fig. 1). ROC curve analyses showed that the 25 species markers selected in the previous step performed well in distinguishing CRC patients from control subjects (AUC = 0.912, Fig. 2c).

### Identification and validation of progressive microbiota gene markers in independent metagenomic cohorts

To identify the progressive microbiota biomarkers for colorectal neoplasia, we constructed discovery cohort 1 (32 CRC, 48 AA, and 49 controls) and discovery cohort 2 (30 CRC, 46 AA, and 50 controls, Fig. 1). We further identified the biomarkers continuously increasing or decreasing from the control to advanced adenoma and then to the CRC group at the genus, species, and gene levels by using the Kruskal–Wallis test. Combining the results of discovery cohort 1 and discovery cohort 2, 6 genera, 21 species, and 277 genes were found to meet the set criteria (see Additional file 2: Tables S5, S6, S7). We defined these markers as ‘progressive microbiota biomarkers’, including *Fusobacterium periodonticum*, *Prevotella nigrescens*, *Streptobacillus moniliformis*, and *Atopobium parvulum* (see Additional file 2: Table S6). Each of the progressive disease gene markers had a KEGG Orthology number (see Additional file 2: Table S7).

To verify the stability of the progressive gene markers, three independent cohorts (616 samples from Shinichi Y, 156 samples from Feng Q, and 140 samples from Andrew T) were used as validation cohorts (see Additional file 1: Fig. S4). Although these cohorts with different sample sizes, they showed similar population characteristics on the genes. For the genes with medium distribution in the population (such as the genes in branch 5, see Additional file 1: Fig. S4), it was reflected that the more serious the disease was, the wider the distribution of genes in the population and the more samples with a high abundance of the genes. As the disease progresses in validation cohorts, the progressive microbiota gene markers show a continuous trend of increasing or decreasing in abundance (see Additional file 1: Fig. S5).

### Functional pathway analysis of progressive microbiota gene markers

**Fig. 3:**
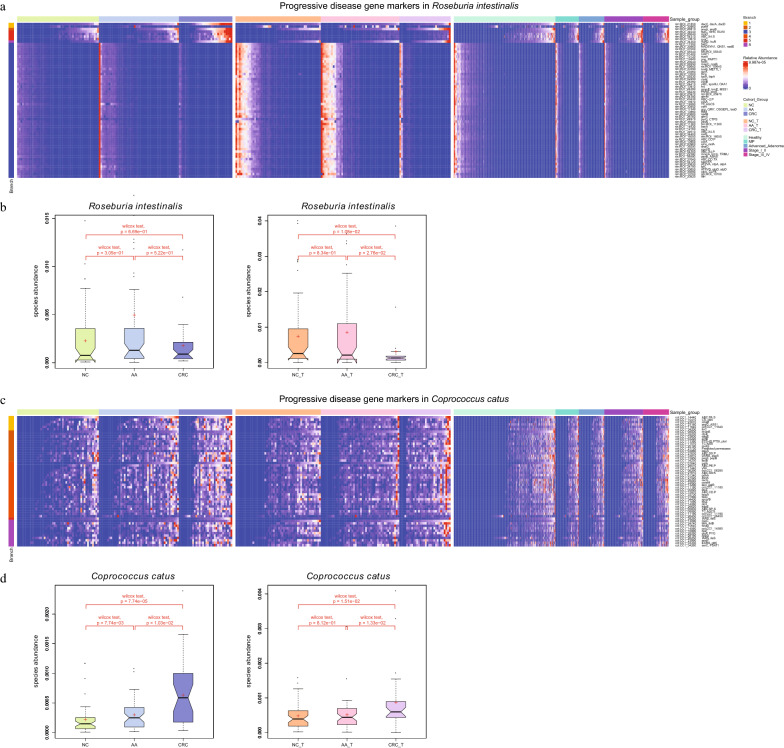
**a** The majority (55/62) of the markers in Roseburia internalis were deceased, while a minority (7/62) of them were increased. **b** The abundances of Roseburia intestinalis in normal controls, AA, and CRC patients. **c** 54 progressive microbiota gene markers were in Coprococcus catus., and all of them were increased. **d** The abundances of Coprococcus catus in normal controls, AA, and CRC patients

61 decreased and 216 increased gene markers were classified as progressive microbiota gene markers. All the 61 decreased markers belonged to six organisms, the majority (90.16%) were from two strains, *Roseburia intestinalis M50/1* (rim: 32 genes) and *R. intestinalis XB6B4* (rix: 23 genes) (Fig. 3a, see Additional file 2: Table S7). A quarter of the 216 increased markers were from one species, *Coprococcus catus* (cct: 54 genes, Fig. 3c). The abundances of Roseburia intestinalis and Coprococcus catus changed slightly along the adenoma-carcinoma sequence (Fig. 3b, d).

To explore the possible mechanism of the progressive microbiota gene markers associated with the development and progression of CRC, we displayed the markers on the metabolic pathways. It was shown that amino acid metabolism, membrane transport, and carbohydrate metabolism contained the most gene markers (see Additional file 1: Fig. S6). For instance, in the sulfur metabolism (map00920) pathway (see Additional file 1: Fig. S7), sulfonate transport system substrate-binding protein (SsuACB, K15554) and homoserine O-succinyltransferase [EC:2.3.1.46] (K00651) were upregulated, and cysteine synthase (cysK, [EC:2.5.1.47], K01738) was downregulated. In taurine and hypotaurine metabolism (see Additional file 1: Fig. S8), phosphate acetyltransferase ([EC:2.3.1.8], K00625) and acetate kinase ([EC:2.7.2.1], K00925) were upregulated. In the cysteine and methionine metabolism (map00270) pathway (see Additional file 1: Fig. S9), homoserine *O*-succinyltransferase ([EC:2.3.1.46], K00651) and homoserine dehydrogenase ([EC:1.1.1.3], K00003) were upregulated.

### Establishment and evaluation of the performance of a novel diagnosis model for colorectal neoplasm

**Fig. 4:**
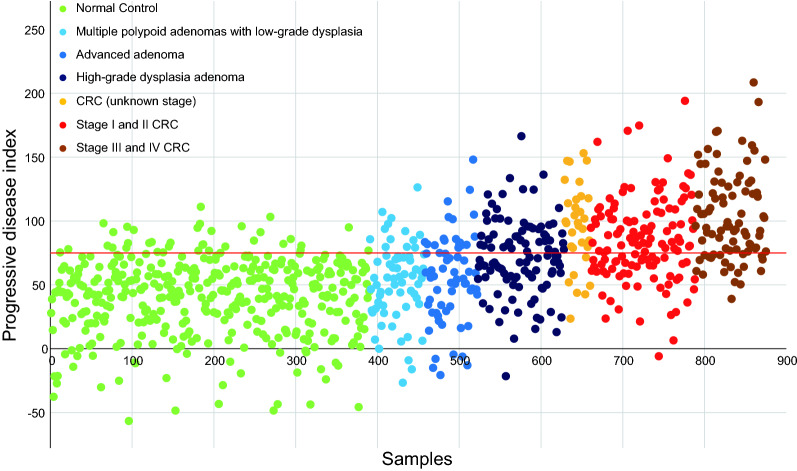
The Progressive Disease Index for the 871 metagenomic samples. 390 samples were control subjects (green), 67 samples were multiple polypoid adenomas with low-grade dysplasia (light blue), 167 samples were advanced adenomas (blue and dark blue), of which 106 were high-grade dysplastic adenoma (dark blue), 247 samples were colorectal cancer patients (orange, red and dark red), of which 128 were stage I/II colorectal cancer (red) and 87 were stage III/IV colorectal cancer (dark red). The Progressive Disease Index increases with the severity of the disease from the perspective of the trend, whose reference warning line was 75

**Fig. 5:**
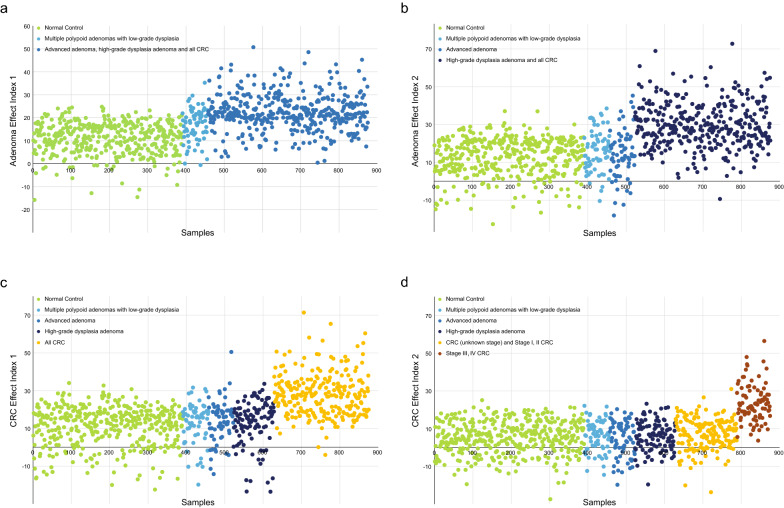
A novel diagnosis model based on the progressive microbiota gene markers with four effect indexes. **a** The value of the AEI1 increased in the advanced adenoma group. **b** The value of the AEI2 increased in the adenoma with the high-grade dysplasia group. **c** The value of CEI1 increased in the CRC group. **d** The value of CEI2 increased in the stage III/IV CRC group

**Fig. 6:**
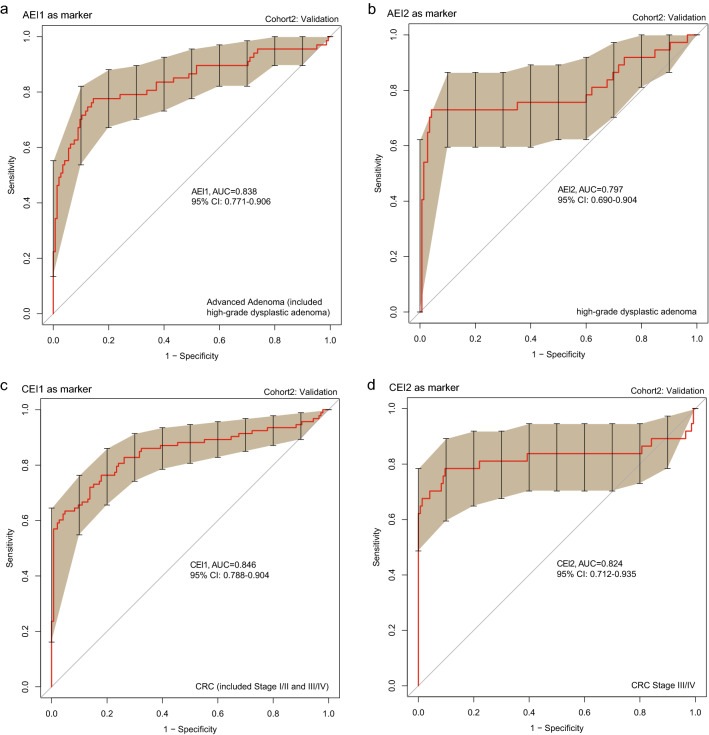
ROC curves of various effect indexes in the validation cohort. **a** ROC curve of Adenoma Effect Index 1 (AEI1) for advanced adenoma. **b** ROC curve of Adenoma Effect Index 2 (AEI2) for adenoma with high-grade dysplasia. **c** ROC curve of CRC Effect Index 1 (CEI1) for all stages of CRC. **d** ROC curve of CRC Effect Index 2 (CEI2) for stage III–IV CRC

We established a diagnosis model based on the progressive microbiota gene markers previously identified. The trends in this model can be quantified using an index score. Overall, the index scores showed a consistently increasing trend from control to advanced adenoma to CRC with different stages (Fig. 4). We further tested the performance of the diagnosis model with four effect indexes in the training cohort (see Additional file 1: Fig. S10) and validation cohort, including the Adenoma Effect Index 1 (AEI1) for AA (Fig. 5a), Adenoma Effect Index 2 (AEI2) for adenoma with high-grade dysplasia (HGD, Fig. 5b), CRC Effect Index 1 (CEI1) for all stages of CRC (Fig. 5c), and CRC Effect Index 2 (CEI2) for stage III-IV CRC (Fig. 5d). The AEI1 showed a sensitivity of 71.3% and specificity of 90.5% for advanced adenoma (Table 1), with an AUC of 0.838 (95% CI 0.771–0.906, Fig. 6a). The AEI2 showed the performance in detecting high-grade dysplasia adenoma with a sensitivity of 76.4% and specificity of 90.3% (Table 1) while achieving an AUC of 0.797 (95% CI 0.690–0.904, Fig. 6b). The sensitivities of the CRC Effect Index 1 for all stages of CRC, and CRC Effect Index 2 for CRC at stage III-IV were 60.3%, and 74.7%, respectively, and the specificities were 94.6%, and 94.6%, respectively (Table 1), with AUCs of 0.846 (95% CI 0.788–0.904) and 0.824 (95% CI 0.712–0.935), respectively (Fig. 6c, d).

**Table 1:** Diagnostic performance for different colorectal neoplasms of a diagnosis model with various effect indexes

Variables	AEI1 (AA)	AEI2 (HGD)	CEI1 (CRC all stage)	CEI2 (CRC III–IV stage)
AUC	0.838	0.797	0.846	0.824
95% CI	0.771–0.906	0.690–0.904	0.788–0.904	0.712–0.935
Sensitivity	71.3% (119/167)	76.4% (81/106)	60.3% (149/247)	74.7% (65/87)
Specificity	90.5% (353/390)	90.3% (352/390)	96.7% (369/390)	96.7% (369/390)

*AA* advanced adenoma, *HGD* high-grade dysplasia, *AUC* areas under the receiver operating characteristic curve, *CI* confidence interval, *AEI1* Adenoma Effect Index 1, *AEI2* Adenoma Effect Index 2, *CEI1* CRC Effect Index 1, *CEI2* CRC Effect Index 2

### Combination with FIT improves the diagnostic ability of the model for CRC

**Fig. 7:**
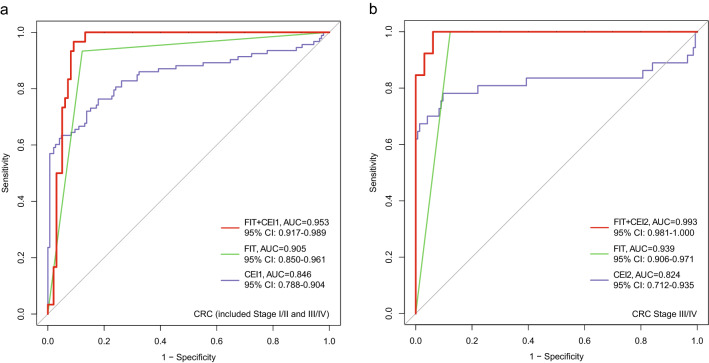
Comparing performances of the CRC Effect Indexes combined with FIT and Indexes or FIT alone in the group of Beijing samples. **a** ROC curve showing the discrimination accuracy of CEI1 + FIT and CEI1 or FIT alone for all stages of CRC. **b** ROC curve showing the discrimination accuracy of CEI2 + FIT and CEI2 or FIT alone for stage III–IV CRC

The FIT was performed in a subgroup of samples from Beijing in this study (30 CRC, 98 adenomas, and 99 controls). The combination of FIT with CEI1 and CEI2 performed significantly superior to Indexes alone in diagnosing CRC, with sensitivities of 96.7% and 100% for all stages of CRC and stage III-IV CRC, while with specificities of 90.8% and 93.9%, respectively (Table 2). Furthermore, the combination achieved AUCs of 0.953 (95% CI 67.90% to 0.989) and 0.993 (95% CI 0.981–1.000), respectively, also better than the FIT alone (AUC = 0.905 for all-stage CRC, AUC = 0.939 for advanced-stage CRC) (Fig. 7a, b).

**Table 2:** Diagnostic ability of a combination of CEI1 and CEI2 with the FIT for CRC

Variables	AUC	95% CI	Sensitivity	Specificity
CEI1+FIT (CRC all stage)	0.953	0.917 to 0.989	96.7% (29/30)	90.8% (89/98)
CEI2+FIT (CRC III-IV stage)	0.993	0.981 to 1.000	100% (13/13)	93.9% (92/98)

*FIT* fecal immunochemical test, *AUC* areas under the receiver operating characteristic curve

## Discussion

In the present study, the most salient finding was that we identified the progressive microbiota gene markers associated with the development of CRC, whose abundance was significantly increased or decreased from normal to adenoma and carcinoma. We further constructed a diagnosis model based on the progressive gene markers and evaluated the application values of which in the non-invasive diagnosis of advanced adenoma and CRC. It was demonstrated that the diagnosis model achieved powerful classification potential for distinguishing advanced adenoma from control subjects with a sensitivity of 71.3%.

Early diagnosis of colonic adenomas and cancer can be recognized as an effective way to strongly reduce the incidence of CRC. Whereas, the current large-scale utilization of non-invasive tests such as the FIT and carcinoembryonic antigen (CEA) tests are limited by low sensitivity [18, 19]. Therefore, novel diagnostic markers for early CRC are urgently needed. The human gut microbiota has been considered the most important ecosystem symbiotic with the body [20–24]. The concept of the gut microbiome serving as a non-invasive diagnosis tool for CRC is a hot research topic in recent years. Yu et al. performed the first metagenomic profiling analysis of CRC fecal microbiomes to identify and validate microbial biomarkers in different cohorts [9]. And growing studies have identified a large number of fecal microbial markers in diagnosing CRC such as Fusobacterium nucleatum (Fn), Fecal clostridium symbiosum, Parvimonas micra ATCC 33270, and Streptococcus anginosus [10, 25, 26]. Dai et al. identified potential diagnostic bacterial markers that are robust across populations and revealed that the comprehensive meta-analysis of shotgun metagenomics data can provide useful functional capacities for CRC-associated microbiota [27]. Due to the molecular markers for adenoma being limited, a novel bacterial gene marker from a Lachnoclostrium sp., labeled as m3 has been identified recently with a sensitivity of 50.8% and 44.2% for advanced and non-advanced adenomas, respectively, which showed promising diagnostic accuracy for adenomas [11]. These emerging pieces of evidence suggest that gut microbiota biomarkers are highly promising and may play a significant role in the detection of CRC in the future.

Here, our study performed shotgun metagenomic-sequenced microbial fecal samples from diverse populations and found bacterial gene markers that were consistently enriched or decreased in advanced adenoma or CRC. The diagnosis model establishment based on the markers yielded strong diagnosis potential for early CRC, which provides a new idea for the ultimate goal would be to identify fecal microbial gene markers to detect colorectal neoplasms. Compared with specific bacteria markers, gut microbiota gene markers can reach a stronger ability for advanced adenomas and are non-affected easily by environmental factors including the difference in ethnicity, geographic regions, and diets because of the validation across independent cohorts. Nevertheless, inconsistent with previous publications, Bifidobacterium which is commonly recognized as probiotics were enriched in patients with AA, while potentially pathogenic Bacteroides and Parabacteroides were enriched in healthy controls in our study. This may be related to the gut microbiota in humans is a diverse and complex micro-ecosystem that is affected by various factors and cannot be answered by our study design. Even so, the previous study has also shown that the Bifidobacterium genus accumulated in ankylosing spondylitis patients [17]. Another study investigated the role of the gut microbiota in the treatment outcomes of patients with metastatic CRC (mCRC) and reported that Lactobacillus and Bifidobacterium species exhibited higher abundance in the progressive disease (PD) group than in the partial response (PR) group [28]. Additionally, some Bacteroides species have been demonstrated to exhibit significantly lower relative abundance in patients with UC than in healthy controls [29]. Thus, it is still deserved to pay significant attention to the gut microbiomes' influence on a host of human diseases.

Changes in the metabolic profile can enhance the production of toxic metabolites to promote the development of CRC. Functional pathway analysis of the progressive microbiota gene markers revealed that sulfonate transport system substrate-binding protein (SsuACB, K15554) was upregulated in sulfur metabolism, phosphate acetyltransferase ([EC:2.3.1.8], K00625), and acetate kinase ([EC:2.7.2.1], K00925) were upregulated in taurine and hypotaurine metabolism, while cysteine synthase (cysK, [EC:2.5.1.47], K01738) was downregulated (see Additional file 1: Figs. S7, S8). All of these could cause the increase of sulfur compounds or H_2_S,, which could ultimately promote the occurrence and development of CRC (see Additional file 1: Fig. S11) [30–32]. Moreover, cysteine synthase (cysK, [EC:2.5.1.47], K01738) was downregulated, which could also reduce the synthesis of l-cysteine (see Additional file 1: Figs. S7, S11). Cysteine is the synthetic precursor of the antioxidant glutathione and intestinal barrier mucin [33, 34]. A reduction in cysteine leads to a reduction in the synthesis of these two substances. A decrease in intestinal wall barrier mucin leads to thinning of the mucosal protective barrier and increases the risk of infection by pathogenic bacteria or harmful substances [34]. It can eventually increase the risk of CRC by increasing levels of vascular cell adhesion molecule-1, cytokines (interleukin-6 and tumor necrosis factor cytokine), and chemokines (high sensitivity C-reactive protein) and inducing the adhesion of T cells and monocytes [35–39]. Therefore, these data suggested progressive microbiota gene markers to be meaningful and highly predictive of CRC status.

To our knowledge, this is the first attempt to establish and validate the diagnosis model for colorectal advanced adenoma and carcinoma based on the progressive gut microbiota gene markers. Functional pathway analysis of these markers showed that certain gene functions were significantly associated with CRC. These findings in the current study could enable future work that aims to precisely determine the contribution of gut microbiota to CRC development and hypothesis-driven mechanistic studies. Despite best efforts, our study had the limitation. As the performance of the diagnosis model was evaluated in these case–control samples, future validation is required in large sample cohorts representative of the CRC screening populations.

## Conclusions

In conclusion, we identified progressive gene markers development along the colorectal adenoma-carcinoma sequence based on the metagenomic analysis of the fecal microbiome. We further reported the successful diagnosis model establishment and cross-region validation for colorectal advanced adenoma and carcinoma, representing a vital step forward in finding a non-invasive, sensitive, and specific diagnostic for colorectal neoplasms.

## Supplementary Information

**Additional file 1: Fig. S1**. Differences in phylogenetic abundance between advanced adenoma patients (red) and control subjects (blue). The phylotypes that were increased (a, c) or decreased (b, d) in the advanced adenoma patients at the genus and species levels. Wilcoxon rank-sum tests were used to identify the differentially abundant genera and species. **Fig. S2. **Linear discriminant analysis (LDA) effect size (LEfSe) analysis revealed the genus difference (a) and species difference (b) in fecal microbiota between the advanced adenoma patients (negative score) and the control subjects (positive score). **Fig. S3. **The Bray-Curtis tree of 49 controls and 48 advanced adenoma patients and 300 disease cases from the previous papers. It was based on the top 30 genus abundance profiling. The 32 CRC samples were all in Hongkong and had the same branch as the 49 controls and the 48 advanced adenoma patients in the Bray-Curtis tree. **Fig. S4**. The relative abundance of the progressive gene markers in the five cohorts. Five cohorts were arranged on the horizontal axis, including discovery cohort 1, discovery cohort 2, validation cohort 1, validation cohort 2, and validation cohort 3. Each cohort has three groups (control group, adenoma group, and CRC group). All 277 progressive disease gene markers were arranged on the vertical axis. **Fig. S5.** The 15 progressive gene markers with decreased abundance (a) and 15 markers with increased abundance (b) were shown in the validation cohort. Some of the median values of the gene abundance were zero, however, the third quartile values of the gene abundance can reflect the expected trend. **Fig. S6.** The increased and decreased progressive fecal microbiota gene markers distributed in the KEGG pathways. **Fig. S7.** The progressive gene markers in the sulfur metabolism pathway. The genes with a red frame were up-regulated from the control group to the advanced adenoma group and then to the CRC group, and the genes with the green frame were down-regulated.** Fig. S8**. The progressive gene markers in taurine and hypotaurine metabolism pathway. The gene with a red frame was up-regulated from the control group to the advanced adenoma group and then to the CRC group.** Fig. S9**. The progressive gene markers in cysteine and methionine metabolism pathway. The genes with a red frame were up-regulated from the control group to the advanced adenoma group and then to the CRC group, and the genes with the green frame were down-regulated. **Fig. S10.** ROC curves of various effect indexes in discriminating patients with advanced adenoma and CRC from control subjects in the training cohort, respectively. (a) The ROC of Adenoma Effect Index 1 (AEI1) for advanced adenoma. (b) The ROC of Adenoma Effect Index 2 (AEI2) for adenoma with high-grade dysplasia. (c) The ROC of CRC Effect Index 1 (CEI1) for all stages of CRC. (d) The ROC of CRC Effect Index 2 (CEI2) for stage III-IV CRC. **Fig. S11. **Schematic diagram of the functions of the progressive microbiota gene markers. The increase of the H2S, decrease in cysteine, and accumulation of homocysteine of the gene markers are related to the occurrence and development of CRC. The biofilm regulator gene was also upregulated in colorectal adenoma and carcinoma, consistent with its roles in CRC carcinogenesis such as destroying the intestinal barrier and dysbiosis.

**Additional file 2: Table S1** The clinical data for the samples in discovery cohort 1 and discovery cohort 2. **Table S2** Clinical data and group information for the 616 samples downloaded from Shinichi Yachida et al. (616 metagenome samples). **Table S3** The statistics of data in discovery cohort 1 and discovery cohort 2. **Table S4** The species biomarkers enriched in advanced adenoma group or normal control group (differential analysis between advanced adenoma patients and normal controls) in discovery cohort 1. **Table S5** The progressive disease genus markers for advanced adenoma and colorectal cancer in gut microbiome in discovery cohort 1 and discovery cohort 2. **Table S6** The progressive disease species markers for advanced adenoma and colorectal cancer in gut microbiome in discovery cohort 1 and discovery cohort 2. **Table S7** The progressive disease gene markers for advanced adenoma and colorectal cancer in gut microbiome in discovery cohort 1 and discovery cohort 2. **Table S8** The key parameters for Adenoma Effect Index 1 (AEI1), Adenoma Effect Index 2 (AEI2), CRC Effect Index 1 (CEI1) and CRC Effect Index 2 (CEI2). **Table S9** The abundance profiling of the progressive disease gene markers in discovery cohort 1 and discovery cohort 2. **Table S10** The cutoff, sensitivity and specificity of score1 and score2.

## Data Availability

Shotgun sequencing data are available from the corresponding author, PJ, upon reasonable request. The data generated in this study are available in NCBI (PRJNA758208). All other data relevant to the study were included in the article or uploaded as Additional files.

## Abbreviations

CRC: Colorectal cancer
NCBI: National center for biotechnology information
PCR: Polymerase chain reaction
FIT: Fecal immunochemical test
CEA: Carcinoembryonic antigen
KEGG: Kyoto encyclopedia of genes and genomes
AJCC: American joint committee on cancer
AUC: Areas under the receiver-operating curve
CI: Confidence interval
HGD: High-grade dysplasia
AEI1: Adenoma effect index 1
AEI2: Adenoma effect index 2
CEI1: CRC effect index 1
CEI2: CRC effect index 2
