# Supplementary material for: A novel promising diagnosis model for colorectal advanced adenoma and carcinoma based on the progressive gut microbiota gene biomarkers
Source: Cell Biosci. 2022 Dec 26;12:208. doi: 10.1186/s13578-022-00940-1 (PMC9791776; doi:10.1186/s13578-022-00940-1)

Supplementary Fig. 1

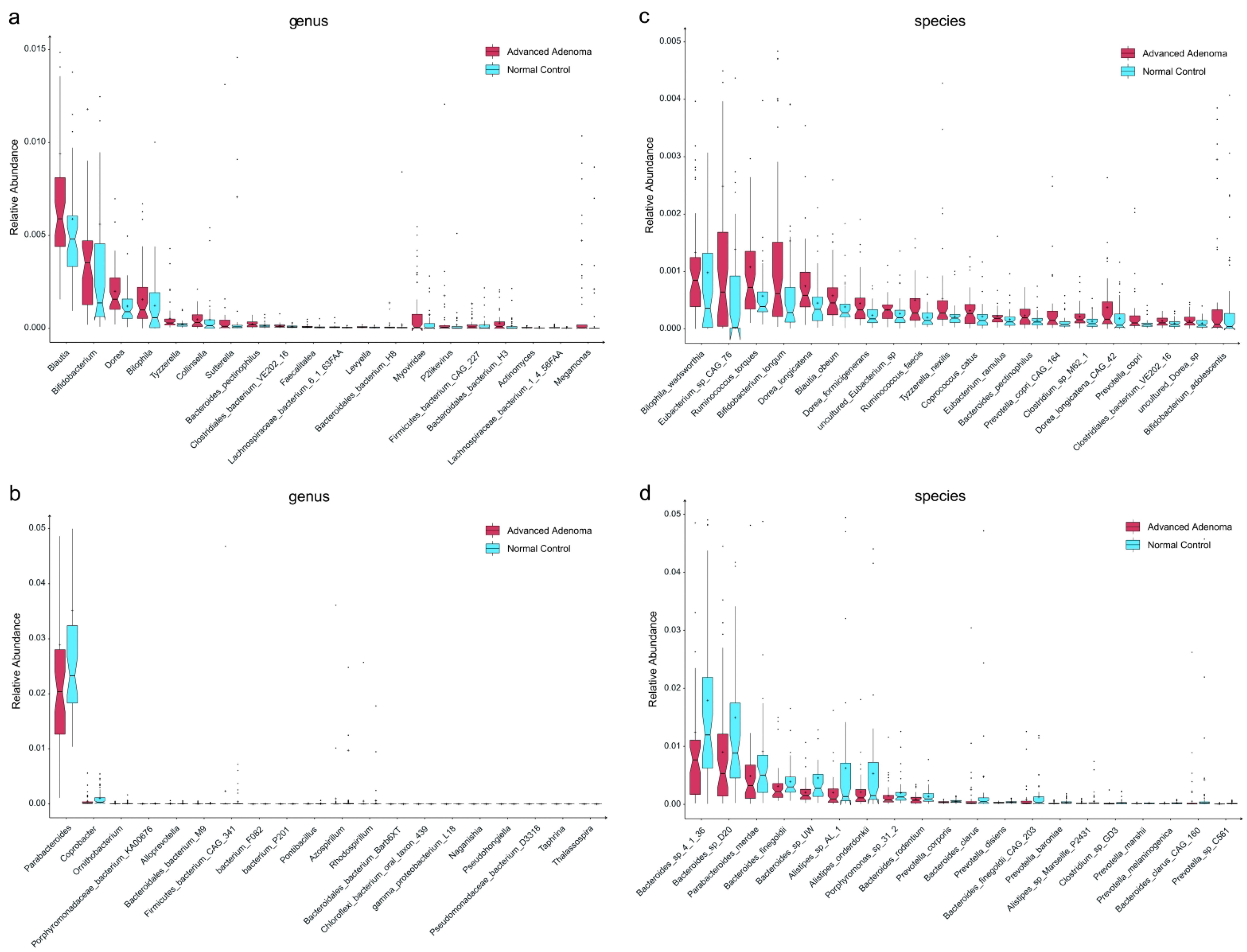

Supplementary Fig. 2

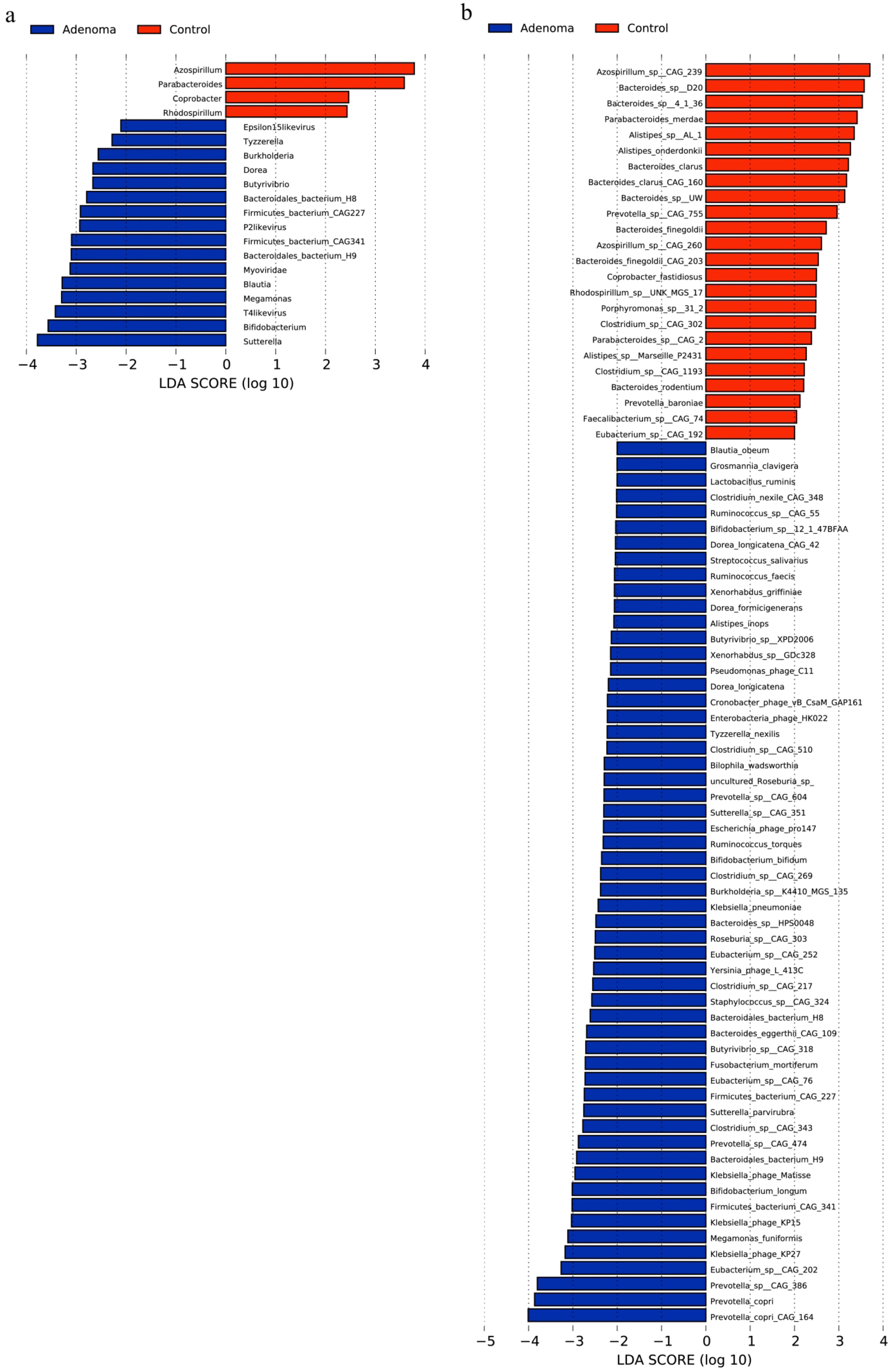

Supplementary Fig. 3

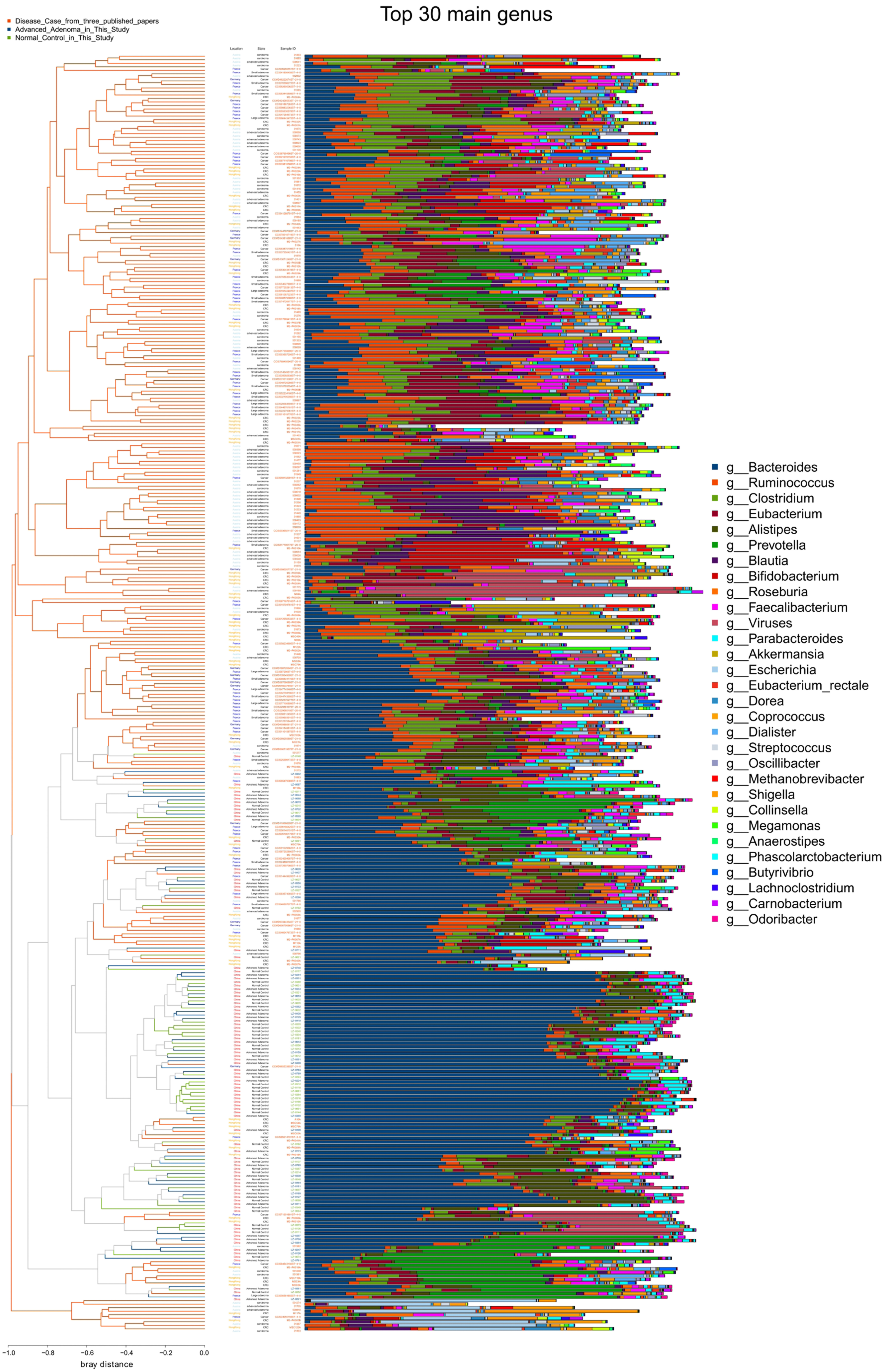

Supplementary Fig. 4

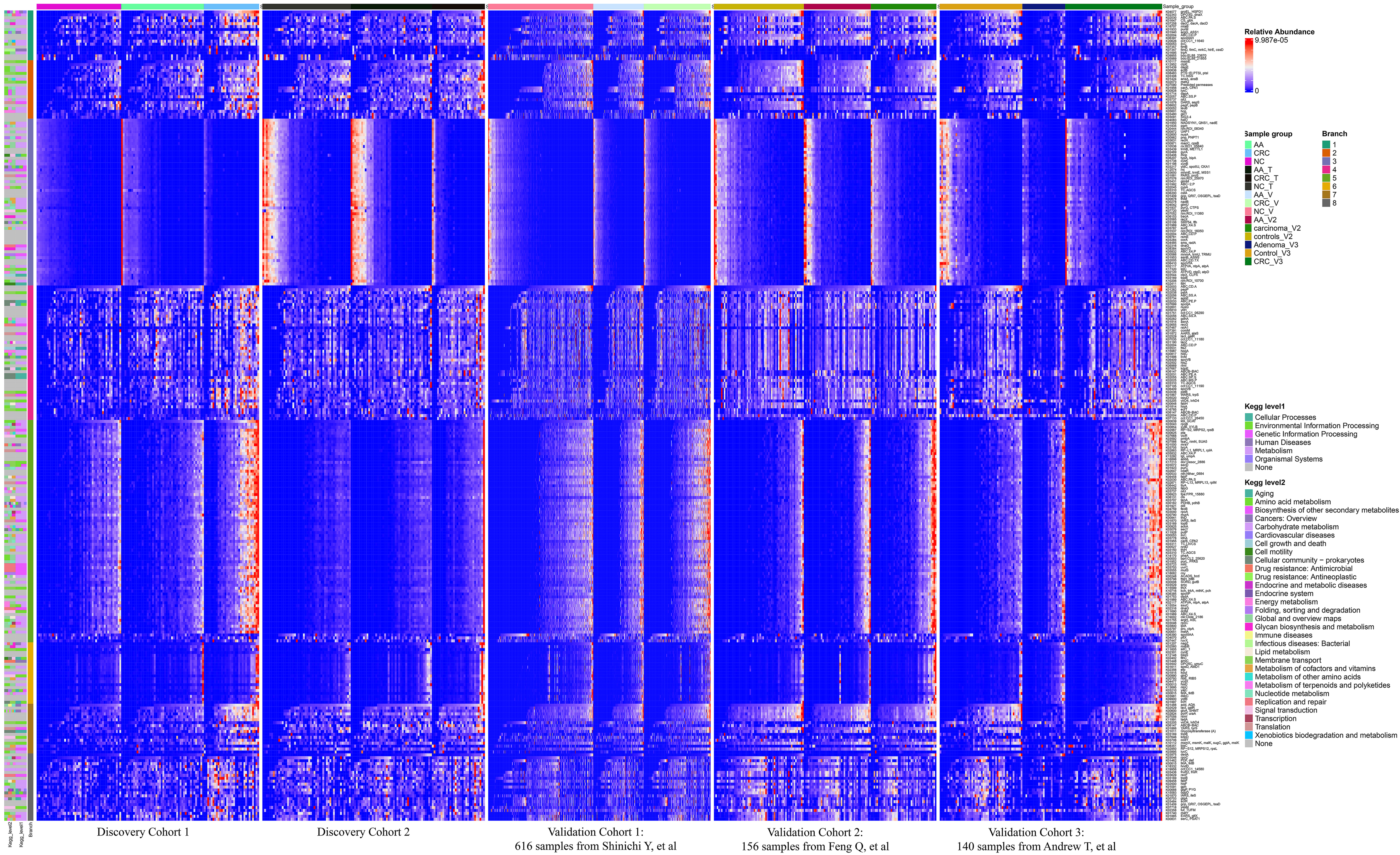

Supplementary Fig. 5

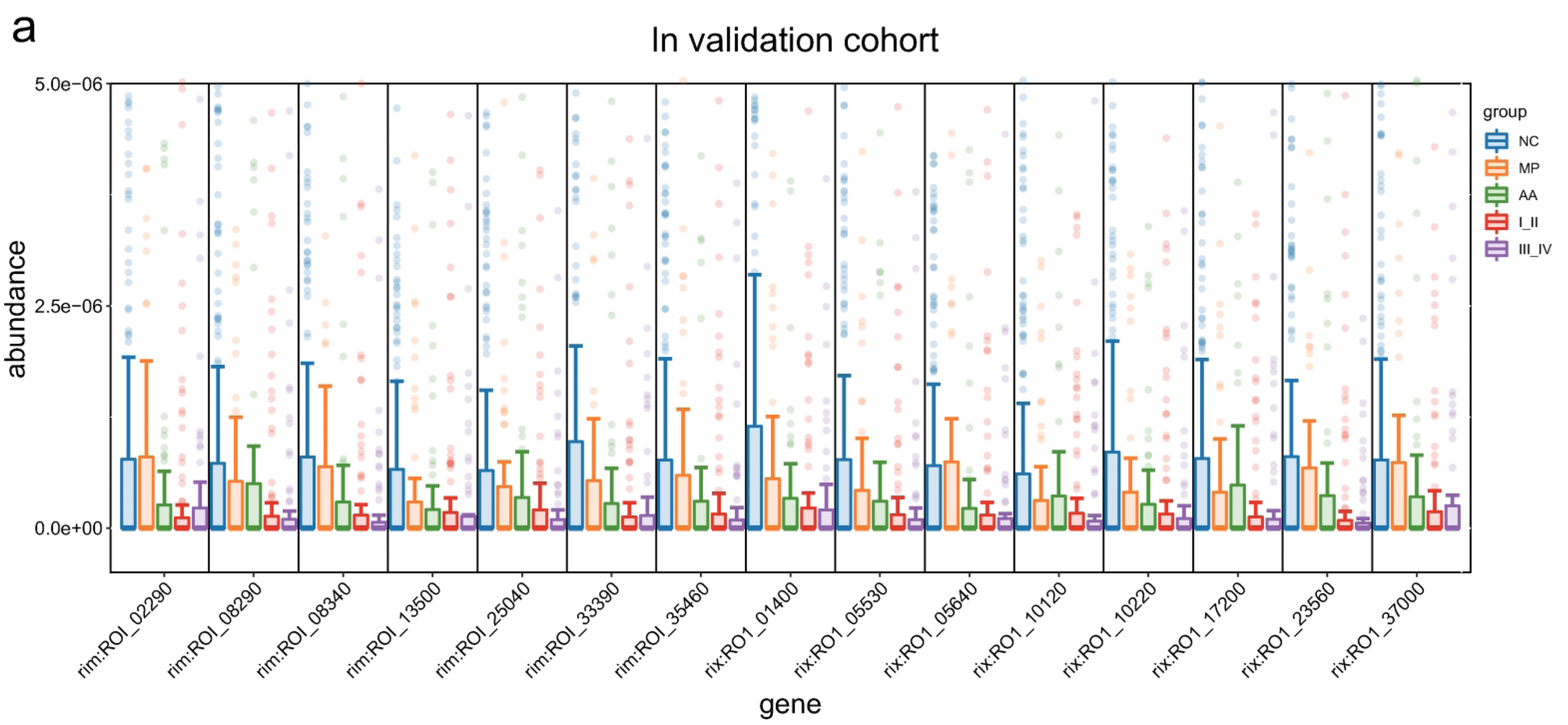

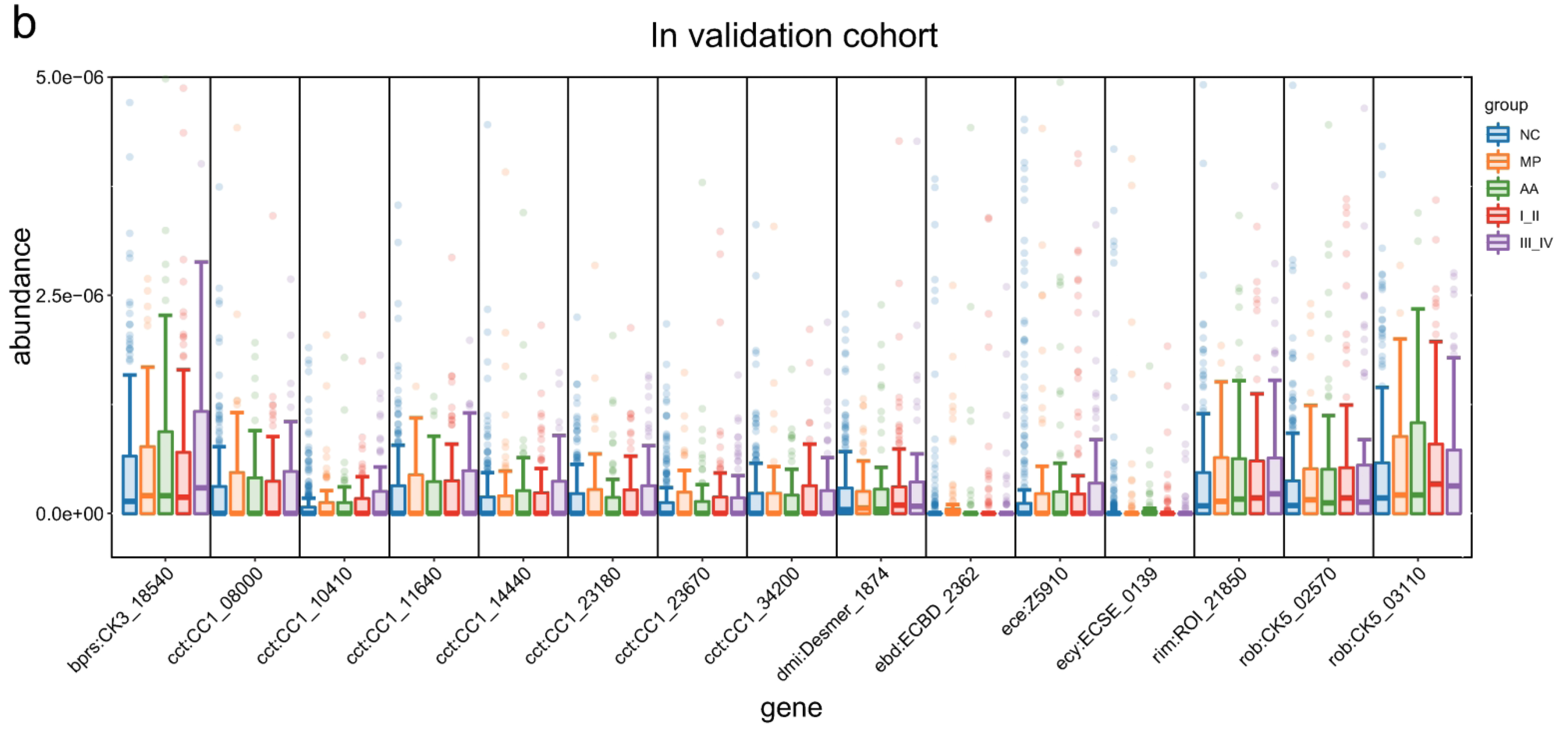

Supplementary Fig. 6

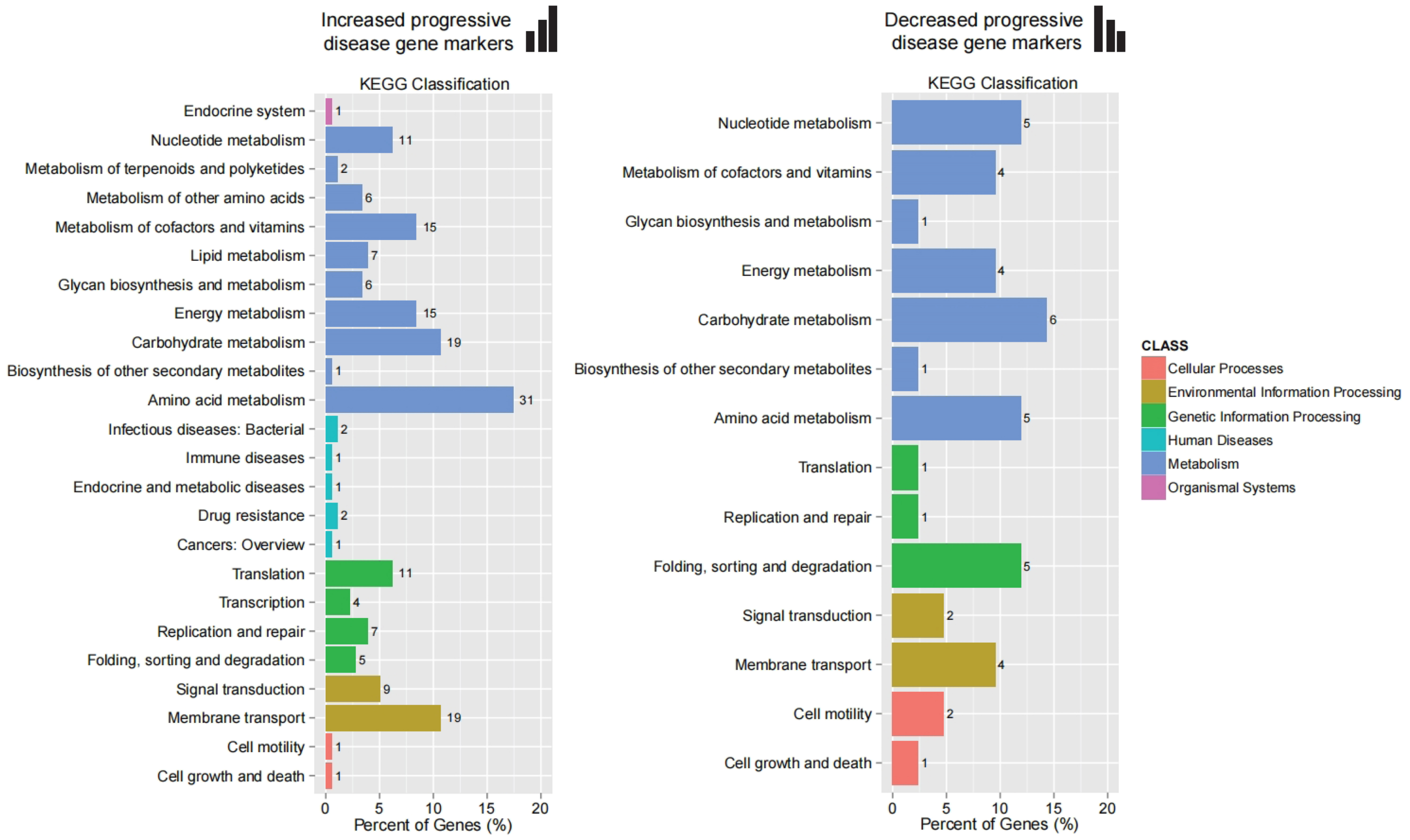

Supplementary Fig. 7

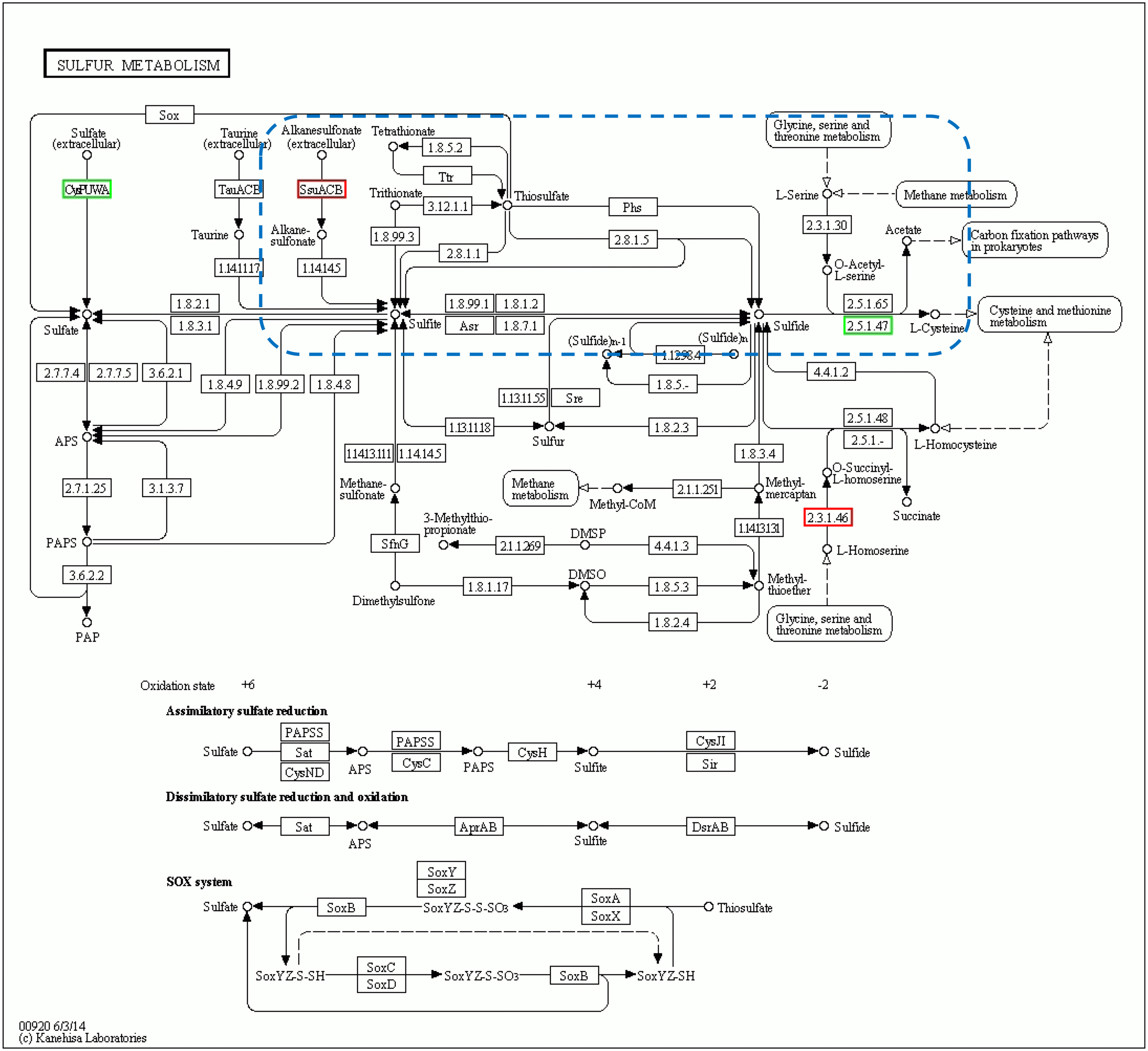

Supplementary Fig. 8

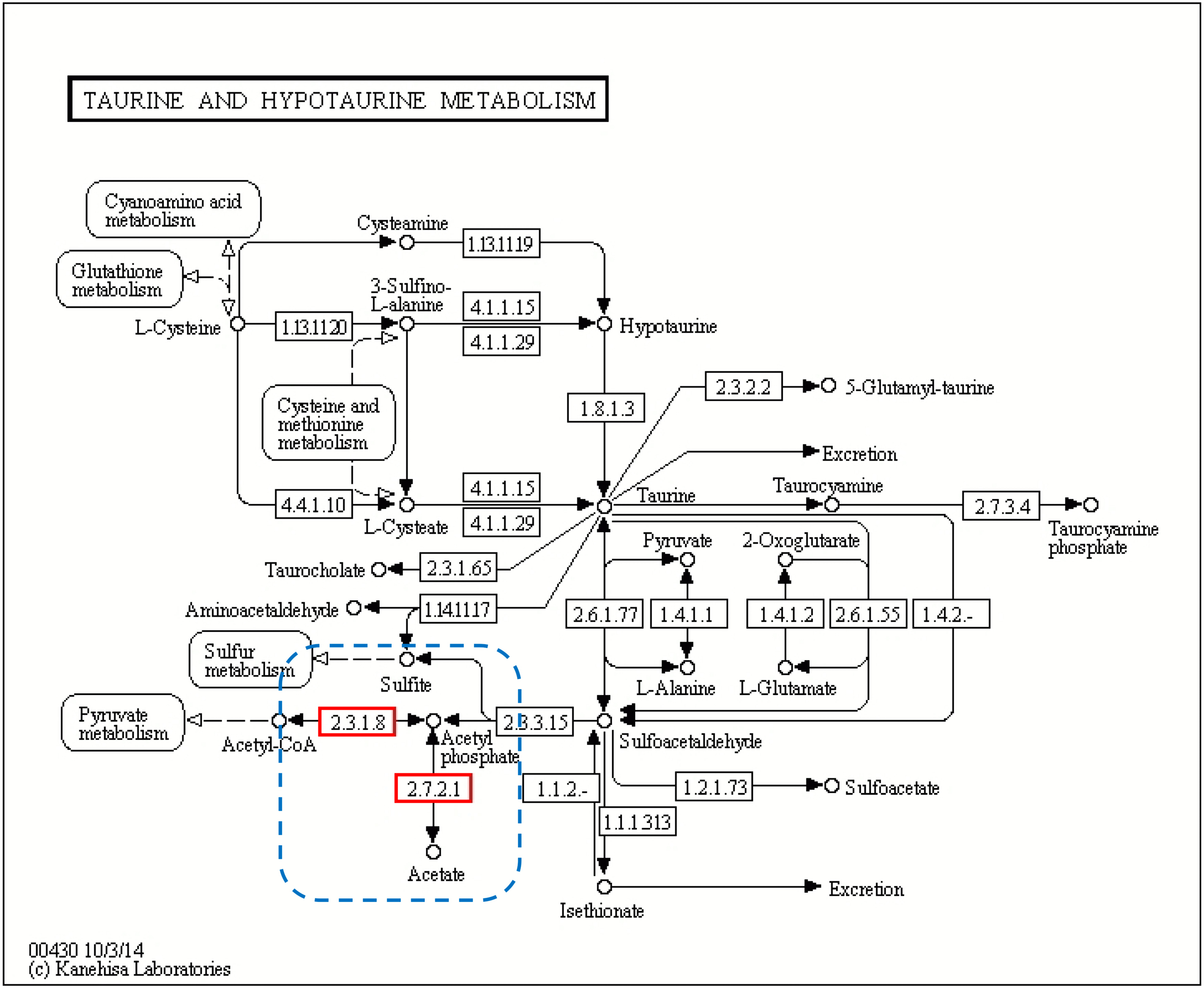

Supplementary Fig. 9

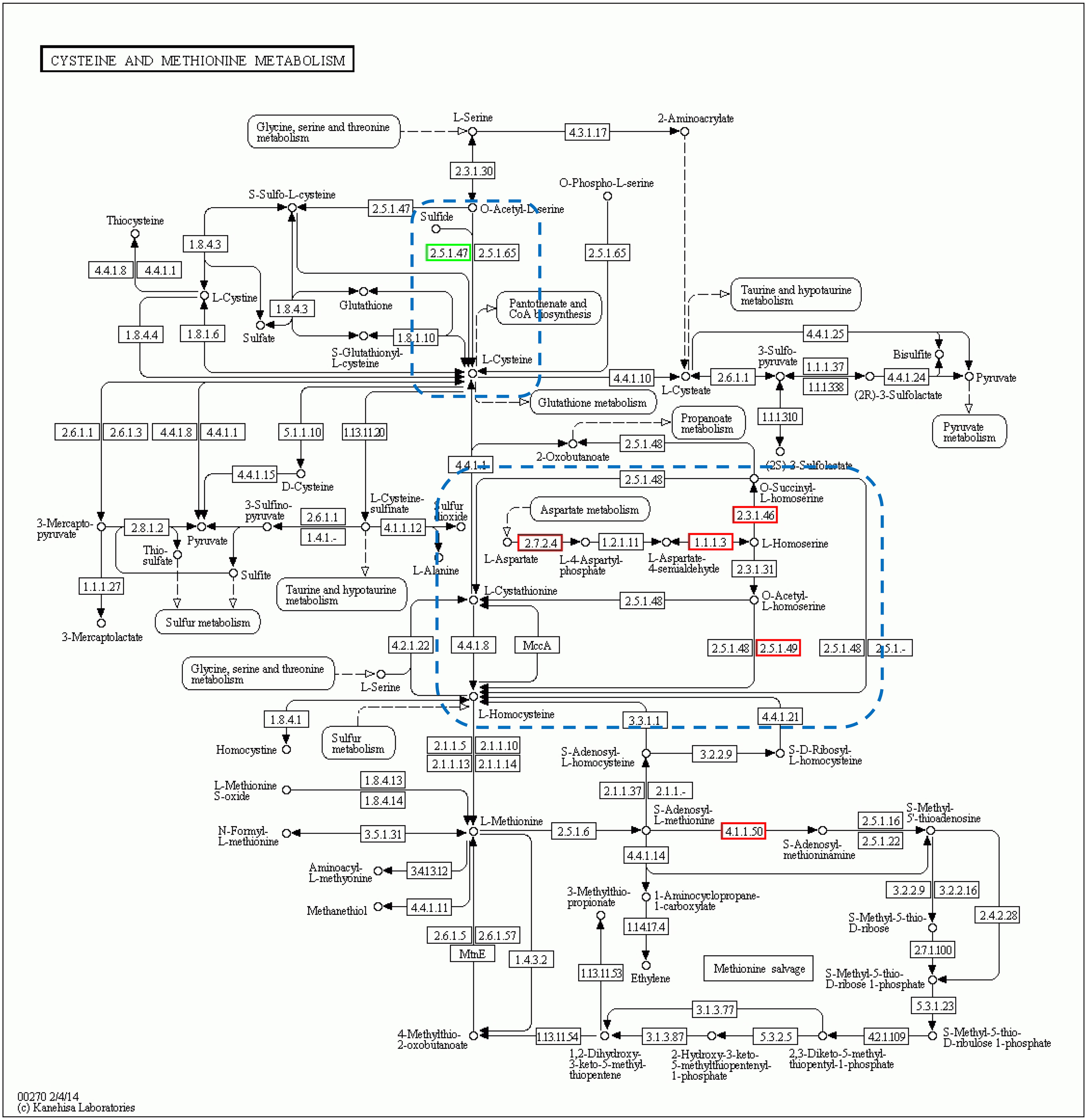

Supplementary Fig. 10

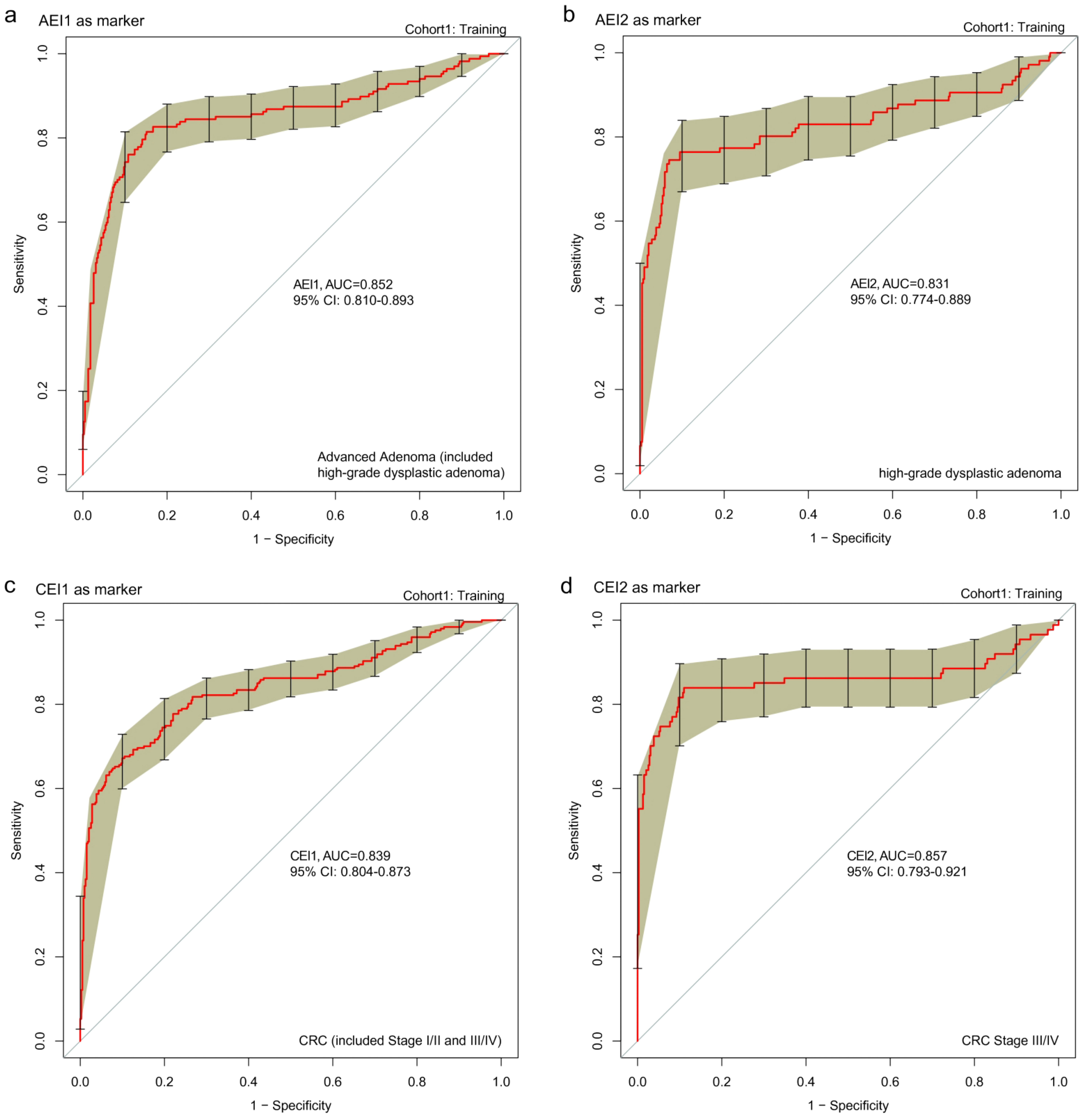

Supplementary Fig. 11

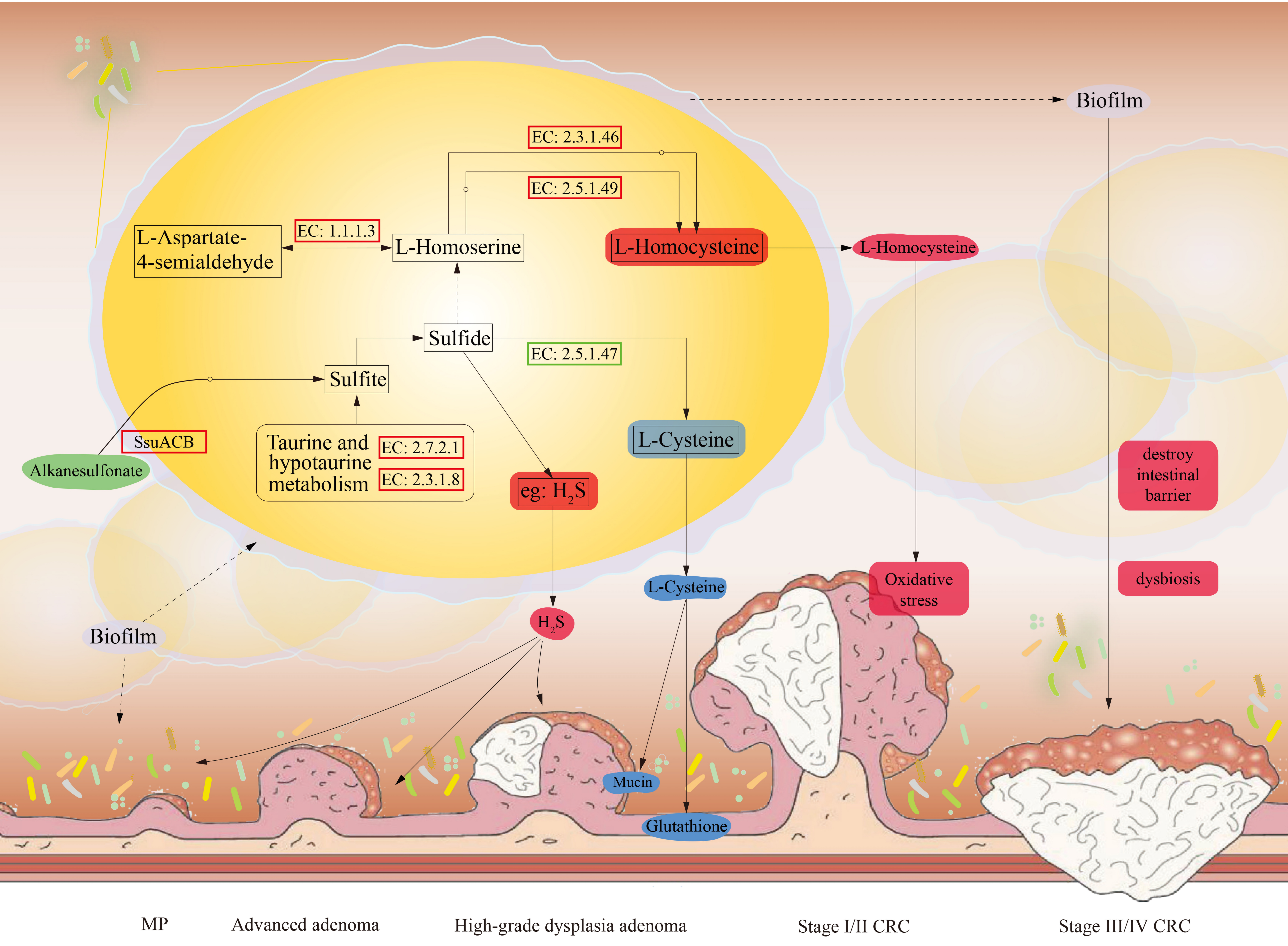

Supplement: Supplementary file 1 — Additional file 1: Fig. S1. Differences in phylogenetic abundance between advanced adenoma patients (red) and control subjects (blue). The phylotypes that were increased (a, c) or decreased (b, d) in the advanced adenoma patients at the genus and species levels. Wilcoxon rank-sum tests were used to identify the differentially abundant genera and species. Fig. S2. Linear discriminant analysis (LDA) effect size (LEfSe) analysis revealed the genus difference (a) and species difference (b) in fecal microbiota between the advanced adenoma patients (negative score) and the control subjects (positive score). Fig. S3. The Bray-Curtis tree of 49 controls and 48 advanced adenoma patients and 300 disease cases from the previous papers. It was based on the top 30 genus abundance profiling. The 32 CRC samples were all in Hongkong and had the same branch as the 49 controls and the 48 advanced adenoma patients in the Bray-Curtis tree. Fig. S4. The relative abundance of the progressive gene markers in the five cohorts. Five cohorts were arranged on the horizontal axis, including discovery cohort 1, discovery cohort 2, validation cohort 1, validation cohort 2, and validation cohort 3. Each cohort has three groups (control group, adenoma group, and CRC group). All 277 progressive disease gene markers were arranged on the vertical axis. Fig. S5. The 15 progressive gene markers with decreased abundance (a) and 15 markers with increased abundance (b) were shown in the validation cohort. Some of the median values of the gene abundance were zero, however, the third quartile values of the gene abundance can reflect the expected trend. Fig. S6. The increased and decreased progressive fecal microbiota gene markers distributed in the KEGG pathways. Fig. S7. The progressive gene markers in the sulfur metabolism pathway. The genes with a red frame were up-regulated from the control group to the advanced adenoma group and then to the CRC group, and the genes with the green frame were down-regula [file 13578_2022_940_MOESM1_ESM.pdf]
